# Music Games: Potential Application and Considerations for Rhythmic Training

**DOI:** 10.3389/fnhum.2017.00273

**Published:** 2017-05-29

**Authors:** Valentin Bégel, Ines Di Loreto, Antoine Seilles, Simone Dalla Bella

**Affiliations:** ^1^Euromov Laboratory, University of MontpellierMontpellier, France; ^2^NaturalPadMontpellier, France; ^3^Homme, Environnement et Technologies de l’Information, Université de Technologie de TroyesTroyes, France; ^4^Institut Universitaire de FranceParis, France; ^5^International Laboratory for Brain, Music and Sound Research (BRAMS)Montreal, QC, Canada; ^6^Department of Cognitive Psychology, Wyzsza Szkola Finansów i Zarzadzania w Warszawie (WSFiZ)Warsaw, Poland

**Keywords:** rhythm, serious game, rehabilitation, movement, training

## Abstract

Rhythmic skills are natural and widespread in the general population. The majority can track the beat of music and move along with it. These abilities are meaningful from a cognitive standpoint given their tight links with prominent motor and cognitive functions such as language and memory. When rhythmic skills are challenged by brain damage or neurodevelopmental disorders, remediation strategies based on rhythm can be considered. For example, rhythmic training can be used to improve motor performance (e.g., gait) as well as cognitive and language skills. Here, we review the games readily available in the market and assess whether they are well-suited for rhythmic training. Games that train rhythm skills may serve as useful tools for retraining motor and cognitive functions in patients with motor or neurodevelopmental disorders (e.g., Parkinson’s disease, dyslexia, or ADHD). Our criteria were the peripheral used to capture and record the response, the type of response and the output measure. None of the existing games provides sufficient temporal precision in stimulus presentation and/or data acquisition. In addition, games do not train selectively rhythmic skills. Hence, the available music games, in their present form, are not satisfying for training rhythmic skills. Yet, some features such as the device used, the interface or the game scenario provide good indications for devising efficient training protocols. Guidelines are provided for devising serious music games targeting rhythmic training in the future.

## Introduction

### Musical Rhythm as a Training Tool

Humans display a natural tendency to move, spontaneously or deliberately, to the beat of rhythmic auditory stimuli, such as music (Repp, 2005; Repp and Su, 2013). This activity is widespread and is typically participatory. It manifests, for example, in dance, synchronized sport, and in group activities (e.g., waving together at a rock concert). Synchronization to a musical beat is sustained by a complex neuronal network, including perceptual regions (the superior temporal gyrus; Thaut, 2003; Chen et al., 2008a; Schwartze and Kotz, 2013), motor regions (e.g., the basal ganglia and the cerebellum; Grahn and Brett, 2007; Chen et al., 2008b; Grahn and Rowe, 2009), as well as sensorimotor integration areas (e.g., premotor cortex; Chen et al., 2006; Zatorre et al., 2007; Kornysheva and Schubotz, 2011). Disruption of these neuronal networks due to brain damage or neurodevelopmental disorder affects auditory-motor synchronization to a musical beat (Corriveau and Goswami, 2009; Bégel et al., 2017), as well as other functions such as speech. For example, difficulties encountered by individuals who stutter in speech production extend to non-verbal sensorimotor skills (Watkins et al., 2008; Falk et al., 2015). In speech production tasks, individual who stutter display reduced activity in brain regions that are also responsible for beat tracking and synchronization to a musical beat such as the basal ganglia (Civier et al., 2013), and the cerebellum (Brown et al., 2005).

Notably, tracking the beat does not require mandatorily an explicit motor response. In perceptual tasks, when detecting a deviation from the beat in an isochronous sequence (anisochrony detection; Ehrlé and Samson, 2005; Dalla Bella et al., 2017b), or detecting if a metronome is aligned or not to the beat (Beat Alignement Task, Iversen and Patel, 2008), the beat is extracted from the auditory signal to perform the judgment. Interestingly, beat extraction in the absence of an explicit motor response recruits motor regions of the brain, such as the basal ganglia (Grahn and Brett, 2007; Grahn and Rowe, 2009), or premotor cortex (Chen et al., 2008a,b). It is worth noting that processing sequences with an underlying beat engages partly separate mechanisms (beat-based timing), as compared to treating single durations (duration-based timing; Coull et al., 2011). The former rely on basal-ganglia-cortical mechanisms, while the latter are more associated to cerebellar-cortical pathways (Grube et al., 2010; Teki et al., 2011). For the purposes of this short review we focus in particular on the training of rhythmic skills engaging beat-based mechanisms.

Because mere listening to an auditory rhythm, for example conveyed via music, activates movement-related areas of the brain, training with rhythmic stimuli may be beneficial to (re)activate the motor system in the damaged and in the healthy brain. There are a few examples of the beneficial effects of rhythm on motor behavior. Rhythmic auditory stimulation can be used as a tool to retrain gait in Parkinson disease (e.g., for increasing speed and stride length; Thaut et al., 1996; Thaut and Abiru, 2010; Benoit et al., 2014; Dalla Bella et al., 2015, 2017a), to improve arm function in stroke (Thaut et al., 1997, 2002, 2007), or to enhance physical performance in sport (e.g., by reducing oxygen consumption in cycling; Hoffmann et al., 2012; Bardy et al., 2015). Positive effects of rhythmic training are not confined to motor behavior, but can extend to perception (Benoit et al., 2014; Dalla Bella et al., 2015). Stimulation using auditory rhythms shows promise also for training speech perception in children with Developmental Language Disorders (e.g., for syntax processing, Przybylski et al., 2013; Schön and Tillmann, 2015). In sum, previous studies point to beneficial effects of a rhythmic training protocol on movement and cognition. In addition, as rhythmic skills are linked to other cognitive abilities such as working memory and reading skills (Tierney and Kraus, 2013; Woodruff Carr et al., 2014), rhythmic training may foster improvements of more general cognitive abilities, which play a critical role in language learning and literacy (Schwartze and Kotz, 2013; Gordon et al., 2015; Kotz and Gunter, 2015). Ultimately, these beneficial effects of rhythmic training are likely to have positive consequences for health and well-being, such as promoting an active lifestyle, by reducing motor and cognitive decline in patient populations or reducing the need for healthcare services.

### Serious Games

A great deal of work over the last two decades has been devoted to devise and promote games for training patients and for rehabilitation. This stream of research has been encouraged by low-cost and widespread new technologies offering unprecedented opportunities to implement training protocols. An increasing number of technologies are designed to improve health and well-being, from smartphone applications to control dietetics (Withings *Wi-Fi Scales*) to movement-based rehabilitation tools using motion capture (Zhou and Hu, 2008; Weiss et al., 2009; Chang et al., 2011). Among them, video games provide a way to entertain people while targeting serious goals, such as the rehabilitation of impaired movement skills (e.g., *Hammer and Planks,* Di Loreto et al., 2013; *Nintendo Wii* games, Saposnik et al., 2010) or cognitive re-entrainment (e.g., *RehaCom,* Fernández et al., 2012) in neurological diseases (for a review and classification of serious games in health, see Rego et al., 2010). In particular, movement-based rehabilitation games exploiting motion capture devices such as the *Wii* or the *Kinect* are a promising way to use technology in the context of re-education (for a review see Webster and Celik, 2014). This method is referred to as “Exergaming”. Note that video games for entertainment may also be used in a serious manner. For example, off-the-shelf video games are often used by physicians for therapeutic purposes, such as *Nintendo Wii* or *Kinect* games (Lange et al., 2009; Barry et al., 2014; Karahan et al., 2015). Exergaming has been proven as efficient in stroke (Webster and Celik, 2014), Parkinson’s disease (Harris et al., 2015), as well as in healthy elderly adults (Sun and Lee, 2013).

During the last 5 years, studies have focused on the cognitive and neuronal underpinnings of the benefits linked to health-targeted serious games (Connolly et al., 2012). On top of the physical and physiological benefits associated with serious games (e.g., via dedicated physiotherapeutic exercises), the effects of this type of training extend to cognition. Cognitive functions such as language and memory can also be enhanced by serious games, an effect which is likely to be accompanied by plastic changes of the brain. For example, structural brain changes associated with learning have been observed due to the use of videogames (Anguera et al., 2013). These promising results indicate that implementing training protocols via serious games may be particularly valuable for enhancing brain functions as well as for therapy and rehabilitation.

In summary, serious games and rhythmic stimulation are promising tools that can be exploited to improve or retrain movement and cognition. We propose that a training of rhythm skills implemented in a serious game would be a means to set up training protocols which may serve rehabilitation of different patient populations. The aim of this review article is to provide an overview of the existing rhythm games and to assess whether they could be well-suited for training purposes. We conducted a survey in which we used criteria such as the precision of the recorded response or the modality of the stimuli provided to evaluate the benefits and limitations of each game.

## Limits and Advantages of The Existing Rhythm Games

To the best of our knowledge, only one music-based training program that uses a game setting has been successfully devised for arm rehabilitation in stroke patients (Friedman et al., 2011, 2014). However, this protocol is not training rhythmic skills *per se* but is rather a music-based adaptation of a standard rehabilitation protocol (i.e., conventional tabletop exercises therapy; Dickstein et al., 1986). To examine whether existing games involving rhythm conveyed by auditory stimuli could be potentially used as training tools, we selected games based on the following inclusion criteria. First, the game has to focus on rhythmic skills. The player must be instructed to synchronize movement (or voice) with stimuli (auditory or visual) which can be predicted on the basis of their temporal structure (i.e., the underlying beat). To our knowledge, no rhythm games currently on the market use purely perceptual tasks, in which the player’s task is to judge the rhythmic features of music. All the games presented below involve movement synchronized to auditory or visual cues. Second, the game device must record the temporal precision of the player’s responses. The scores, levels, difficulty and feedback given to the player must depend on her/his temporal precision in performing the movement. Once the games were selected, they were categorized by: (a) the peripheral used to capture and record the response; (b) the type of response that is recorded; and (c) the output. The peripheral is important to judge if the game is readily usable for training (e.g., in a clinical context). In addition, note that most of the studies in cognitive psychology of rhythm use finger tapping, since this is a simple and objective way to study rhythmic skills (Repp, 2005; Repp and Su, 2013), but other reponses are possible (e.g., full-body motion). Finally, the output is relevant as it indicates whether the games provide a feedback (an outcome measure or score) on the precision of the performance reflecting a participant’s rhythmic skills. These categories are helpful for evaluating the therapeutic potential of each game. For example, a game requiring finger tapping is likely to have a different effect on behavior than a game requiring full body motion, such as dance.

Twenty-seven games on a variety of devices (Wii, PlayStation, PC, Tablet/Smartphone, Xbox, Gameboy) fulfilling the aforementioned criteria were considered for the analysis (see Table 1). These games were classified in four categories as indicated below.

**Table 1:** **List of the reviewed rhythm-based games**.

Game	Peripheral	Type of response recorded	Output
Dance revolution/dancing stage	Dance pad (PS2, PC)	Impacts of feet (PS2)/fingers (PC)	Incrementing score
Donkey konga	Bongos	Impacts of hands	Incrementing score
Dancing with the star	Wiimote, Nunchuk (Wii), keyboard (PC)	Hands movement (Wiimote), Key tapping (PC)	Incrementing score
DJ hero	Turntable replica (Wii, PS 2 and 3, Xbox 360)	Hands and fingers movement on the Turntable	Incrementing score
Everyone sing	Microphone (Wii, PS 3, Xbox 360)	Voice	Incrementing score
Guitar hero	Guitar replica, joystick (Wii, PS 3, Xbox 360), keyboard (PC), screen (tablet, Androîd)	Left-hand key tapping, right-hand key moving up and down (Wii, PS3, Xbox 360), screen tapping (tablet), joystick button pressing (Wiimote, pS3, Xbox 360)	Incrementing score
Just dance	Wiimote (Wii), PS camera, PS move (PS4, PS3), Kinect (Xbox 360, Xbox one)	Hand movement (Wiimote), all-body movement (PS move, PS camera, Kinect)	Incrementing score
Rhythm paradise (USA: Rhythm Heaven Fever)	Nintendo DS, Wiimote	Finger tapping on the screen, hand movement (stylus; DS), key tapping (Wii)	Incrementing score
Rock band	Guitar, Drums replica, Microphone (Wii, Xbox 360, PS3), Tactile screen (Iphone, Ipod Touch), Nintendo DS, PSP	Left-hand key tapping, right-hand key moving up and down (mediator-like), feet impact (bass drum), drumsticks impact (Wii, PS3, Xbox 360, Nintendo DS, PSP), screen tapping (Iphone, Ipod), joystick button pressing (Wiimote, pS3, Xbox 360), voice (microphone)	Incrementing score
140	Keyboard (PC)	Key pressing	Progression in a level
Osu	Mouse (PC)	Key pressing	Incrementing score
Beatmania	Turntable replica (Arcade, PS1, PS2), Nintendo gameboy	Hands and fingers movement on the Turntable (Arcade, PS1, PS2), key pressing (Gameboy color)	Incrementing score
Patapon	PSP	Key tapping	Progression in a level
Rhythm cat	Tablet, Smartphone	Screen tapping, holding, swiping	Incrementing score
Groove coaster zero	Tablet, Smartphone	Screen tapping, holding, swiping	Incrementing score
Igobeat	Tablet, Smartphone	Screen tapping, holding, swiping	Incrementing score
Beat brite	Tablet, Smartphone	Screen tapping, holding, swiping	Incrementing score
Online PC games	Keyboard	Screen tapping	Progression in a level/Incrementing score

*The last row concerns online PC games (available at www.musicgames.co/games-by-category/rhythm-games/) having similar characteristics*.

### Games that Involve Full Body Movements Recorded via an External Interface (e.g., Kinect, Wii)

Here we refer mostly to dance games (e.g., *Just Dance*). These games have interesting applications in physiotherapy for patients with spinal cord injury, traumatic brain injury and stroke (Lange et al., 2009). They focus more on physical exercise and activity than on rhythm *per se*. Indeed, the ability of these games to record and score the rhythmic precision of the player is rather poor. Because these games focus on discrete movements/actions instead of repeated movements (i.e., rhythmic) they cannot be used for delivering specific training of rhythmic skills. For example, *Just Dance* consists in reproducing movements that are illustrated through images displayed on the screen. The player’s score depends on the precision of the movements as compared to a model action sequence. The player has to execute the movements in a given temporal window. Yet, the task is not purely rhythmic and synchronization to the musical beat is not recorded. In spite of the fact that these games do not measure rhythmic skills *per se*, they provide a motivating setting to perform dance while monitoring the player’s movements. Adding a rhythm component to some of these games, as in the case of dance, may be a valuable strategy to translate them into a training program.

### Games that Involve Rhythmic Finger Tapping on a Tablet

An example of these games is *Beat Sneak Bandit*. Here, the player has to tap precisely to the beat in order to make the character progress, avoid the enemies and so forth. This kind of feature is used in serious games dedicated to learning, such as *Rhythm Cat*, designed to learn music rhythm notation. For the purposes of training rhythmic skills, one major drawback of these games is that the timing precision of the software is very poor. The time window in which a response is considered as good is very wide (i.e., up to several hundreds of milliseconds) and the temporal variability of the recording is high. In addition, no feedback on the rhythmic performance of the player is provided.

### Computer or Console Games that Involve Finger Tapping on Keys

These games can be played on a keyboard, using a joystick, or on special devices. One of the most famous is *Guitar Hero*. In this game the player plays on a guitar replica with five keys, and has to push the keys in correspondence of images presented on a screen. Rhythm precision of the responses is recorded and used to compute a performance score. The response must appear in a specific temporal window to be considered as good. The same concept is used in many PC games, but keyboards key (e.g., arrows) are used instead of guitar replica. As in the case of tablet games, the main weakness of these games is their low temporal precision in recording rhythmic performance (around 100 ms in *Guitar Hero*). Nevertheless, these games are interesting as they represent a good starting point to develop serious-game applications aimed at training rhythmic skills.

### Console Games Involving Singing

In these games, the player is asked to sing in synchrony with the music. This is not a rhythmic task *per se*, but the performance involves a rhythmic component. As in classical karaoke, lyrics are presented on the screen. In this case, a feedback (score) is provided to the player while she/he sings and a final global score is given at the end of the performance, including temporal precision (the response must appear in a given temporal window to be considered as good) but also pitch precision. Here, the potential benefit for health rests upon the fact that singing is a good way to restore speech abilities (e.g., fluency) in aphasia following stroke (for example, see Norton et al., 2009).

Even though some of the aforementioned games present good ground for training rhythmic skills, their main drawback is that their temporal precision when recording movement relative to the beat is rather poor. Thus, the output measures provided by these games are insufficient to isolate rhythmic features of the performance (e.g., variability of the motor performance, precision of the synchronization with the beat, etc.). Moreover, in none of these games the rhythmic complexity of musical stimuli has been manipulated. Difficulty is manipulated only through the amount of responses required during the game (e.g., number of visual tags which the player has to react to) which is not a rhythmical feature. For example, using music with various degrees of beat saliency would allow introducing rhythm-based difficulty levels in the game. This has the advantage that rhythms with increased complexity could be presented progressively throughout the game, thus potentially leading to improved beat-tracking skills.

## Conclusion

We reviewed 27 rhythm-based games already in the market that could be used in a rhythmic training protocol. Unfortunately, based on our criteria, none of the aforementioned games is satisfying for this purpose. First, in most of the games, the task consists in reacting to visual stimulations while music is presented. Thus rhythmic skills are not selectively trained. Second, the number of stimuli, instead of the rhythmic characteristics of the music, is varied to change the difficulty of the game. Third, in spite of the fact that the regularity of rhythmic patterns can influence the performance in the game, the response provided by the player is not targeted at the rhythmic aspects of the stimuli. For example, the player touches the screen at the right moment to catch objects or makes full-body movements to imitate model-actions in dance games. In addition, note that the reviewed games do not offer opportunities for controlled functional movement training. For example, none of them provide a guidance to achieve desired movement trajectories. This problem may be overcome in the future by providing relevant feedback when the player approaches optimal movement trajectories (e.g., via sonification, Effenberg et al., 2016). The tasks implemented in these games are vaguely reminiscent of implicit timing tasks (Lee, 1976; Zelaznik et al., 2002; Coull and Nobre, 2008). Explicit and implicit timing have been treated as relatively independent processes in cognitive neuroscience (Zelaznik et al., 2002; Coull and Nobre, 2008; Coull et al., 2011). The former is associated with tasks requiring voluntary motor production (e.g., synchronized tapping tasks; Repp, 2005; Repp and Su, 2013) or overt estimation of stimulus duration (e.g., duration discrimination; Grondin, 1993). In contrast, implicit timing is tested with tasks unrelated to timing (e.g., avoiding a vehicle when crossing the road), but in which temporal prediction affects the performance (judging the time before the vehicle reaches us; Lee, 1976; for more details, see Nobre et al., 2007; Coull and Nobre, 2008; Coull, 2009). In particular, temporal prediction fostered by a regular temporal pattern (e.g., a metronome) of sensory stimuli improves performance in non-temporal tasks (e.g., working memory, Cutanda et al., 2015; pitch judgment, Jones et al., 2002; language judgments, Przybylski et al., 2013).

Despite the available music games are not explicitely targeted at rhythmic training, they may still foster training timing implicitly, in combination with other more explicit processes (e.g., focusing on spatial and pitch accuracy). There is evidence that the implicit dimension of timing may be more robust than explicit timing, for example in beat deafness (Bégel et al., 2017). It is possible that participants with timing disorders (e.g., Parkinson’s disease or developmental stuttering; Grahn and Brett, 2009; Falk et al., 2015) may still be able to capitalize on partly spared implicit timing functions to re-learn rhythmic skills via a training program. Note, however, that so far beneficial effects of rhythm-based training protocols typically made use of explicit timing tasks (e.g., walking with an auditory rhythm; e.g., Lim et al., 2005; Spaulding et al., 2013; Benoit et al., 2014). This may suggest that tasks which recruit explicit timing mechanisms may be a particularly good candidate to build a successful protocol for rhythmic training. In only one of the reviewed games (*Beat Sneak Bandit*), the goal was to tap to the beat of music, which is an explicit timing task. In sum, almost all of the reviewed games do not require participants to perform explicitly rhythmic tasks. Yet, they are likely to engage implicit timing mechanisms. Whether training rhythm implicitely in the context of a music game can lead to positive effects comparable to those found with explicit rhythmic tasks deserves further enquiry.

In summary, the games currently on the market, albeit they are not optimal for rhythmic training, provide at least interesting ideas that might pave the ground to devise successful training programs. Games on portable devices (e.g., tablets or smartphones) using tapping to the beat provide the simplest solution to implement a rhythm training protocol. They are low-cost while offering a motivating and user-friendly environment to train rhythmic skills with a playful interface. Although this solution has some potential, there are two problems. The precision of movement recording relative to the beat, and the ensuing measures of rhythm precision, need significant improvement. To deal with these issues, methods used to analyze synchronization to the beat in the neurosciences of rhythm (Kirschner and Tomasello, 2009; Pecenka and Keller, 2009; Woodruff Carr et al., 2014; Dalla Bella et al., 2017b) should be applied to games designed for rhythm training. In addition, estimations of timekeeper and motor implementation variance (e.g., based on tapping performance) might allow to refine the feedback on the performance (e.g., Schulze and Vorberg, 2002; for a review see, Wing, 2002). This will ensure that a precise feedback on the rhythmic performance can be provided and that the stimuli and game progression can be tailored to individual learning curves. Moreover, to ensure that the training program specifically targets rhythmic skills, stimulus (or response) will have to be varied in terms of rhythmic difficulty. This can be achieved, for example, by selecting musical excerpts based on their rhythmic complexity. Using stimuli with increasing difficulty in beat tracking (e.g., with a less salient beat) throughout the game might allow to progressively fine tune the player’s rhythmic skills. These guidelines should be taken into account in the future to devise efficient protocols for training rhythmic skills via serious music games.

## Author Contributions

VB and SDB conceived the study; VB conducted the survey; VB, IDL and AS contributed to data analysis; all authors contributed to the writing of the manuscript.

## Conflict of Interest Statement

The authors declare that the research was conducted in the absence of any commercial or financial relationships that could be construed as a potential conflict of interest.
